# *Scn2a*, encoding Na_*V*_1.2 channel, contributes to tonotopic maturation of spike kinetics in developing mouse MNTB

**DOI:** 10.3389/fncel.2026.1819425

**Published:** 2026-06-16

**Authors:** Jorge Contreras, Han-Gyu Bae, Jun Hee Kim

**Affiliations:** 1 Department of Otolaryngology-Head & Neck Surgery, Kresge Hearing Research Institute, University of Michigan Medical School, Ann Arbor, MI, United States; 2 Department of Cell and Developmental Biology, University of Michigan Medical School, Ann Arbor, MI, United States

**Keywords:** auditory brainstem, MNTB, *SCN2A*, sodium current, tonotopy

## Abstract

**Introduction:**

*SCN2A*, encoding the voltage-gated sodium channel Na_*V*_1.2, is a high-risk gene associated with autism spectrum disorder (ASD) and has been linked to sensory hypersensitivity. Recent work indicates that Na_*V*_1.2 loss-of-function produces developmental and compartment specific alterations in neuronal signaling. However, how *SCN2A* contributes to the maturation of subcortical auditory circuits that demand exceptional temporal precision remains unclear.

**Methods:**

In this study, using *Scn2a* haploinsufficient (*Scn2a^+/–^*) mice, we investigated the functional contribution of Na_*V*_1.2 to spike-generating mechanisms in the medial nucleus of the trapezoid body (MNTB), a fast inhibitory relay in the auditory brainstem organized along a medial–lateral tonotopic axis.

**Results:**

In the pre-hearing period (P4–P6), *Scn2a* haploinsufficiency reduced transient Na^+^ current amplitude and eliminated a delayed onset inward Na^+^ current component observed in a subset of wild type neurons, providing functional evidence for Na_*V*_1.2 dependent activity in developing MNTB neurons. Notably, Na_*V*_1.2 dependent deficits were tonotopically patterned. Lateral (low frequency) MNTB neurons exhibited the largest reductions in both transient Na^+^ current and persistent Na^+^ current, whereas medial neurons were comparatively spared in peak current magnitude. In current clamp, *Scn2a^+/–^* neurons displayed altered action potential kinetics during the pre-hearing window (slower and broader spikes), but repetitive firing during prolonged depolarizing steps was largely preserved, indicating that *Scn2a* reduction impacts spike waveform maturation more than tonic spike count. After hearing onset, peak Na^+^ current amplitudes were comparable between genotypes (P14-P24), consistent with developmental reorganization of Na_*V*_ channel contributions.

**Discussion:**

Together, these findings identify a pre-hearing, tonotopically biased role for *Scn2a* in axon initial segment (AIS)-linked Na^+^ channel function and spike kinetics in the MNTB, providing a mechanistic framework for how *Scn2a* may influence early auditory brainstem development relevant to sensory phenotypes in ASD.

## 1 Introduction

Autism spectrum disorder (ASD) is a neurodevelopmental disability characterized by social communication, repetitive behaviors, or sensory processing impediments (American Psychiatric Association [APA], 2022). Among individuals with ASD, auditory deficits such as reduced tolerance to sounds and auditory processing difficulties are commonly reported even when peripheral hearing is normal (O’Connor, 2012). These observations motivate molecular and cellular approaches to understand how auditory pathways encode sound with exceptional speed and precision. *SCN2A*, which encodes the voltage-gated sodium channel Na_*V*_1.2, is a high-risk gene for ASD (Sanders et al., 2018). Consistent with *SCN2A* role in sensory processing, *SCN2A* loss-of-function disrupts cerebellar plasticity and hypersensitizes sensory reflexes, providing a mechanistic link between Na_*V*_1.2 dysfunction and sensory hypersensitivity in *SCN2A*-associated ASD (Wang et al., 2024). A recent study emphasizes that Na_*V*_1.2 loss-of-function produces developmental and compartment specific effects on neuronal signaling (Spratt et al., 2021). *Scn2a* haploinsufficiency disrupts dendritic excitability and synaptic integration in cortical pyramidal neurons, thus, *SCN2A* can alter information processing in a circuit-dependent manner (Spratt et al., 2021). However, it is unknown whether *SCN2A* haploinsufficiency perturbs the maturation of Na_*V*_1.2 channel function and spike generation in the developing auditory nervous system.

The medial nucleus of the trapezoid body (MNTB) is one of key auditory brainstem nuclei in binaural auditory circuits. The MNTB principal neurons receives contralateral excitatory input from globular bushy cells and converts this giant glutamatergic input from the calyx of Held into precisely timed inhibitory output with exceptionally high temporal fidelity (Kuwabara et al., 1991; Smith et al., 1993; Schneggenburger and Forsythe, 2006; Borst and Soria van Hoeve, 2012). Electrophysiological studies have shown that MNTB principal neurons express large, fast transient inward Na^+^ currents that support rapid spike initiation and are reliable during high-rate synaptic drive (Forsythe and Barnes-Davies, 1993; Ming and Wang, 2003). At the calyx–MNTB synapse, Na^+^ channel availability and recovery from inactivation are key determinants of high-frequency firing. Moreover, developmental remodeling of presynaptic Na^+^ channel expression/properties contributes to maturation of high-fidelity transmission (Leão et al., 2005; Kim et al., 2010). More broadly, spike initiation and propagation depend on dense clustering of voltage-gated Na^+^ channels at specialized axonal domains (e.g., the AIS of MNTB neurons or the heminode of the calyx terminal) (Berret et al., 2016; Xu et al., 2017; Kim et al., 2019). During CNS maturation, Na^+^ channel clustering transitions from Na_*V*_1.2, encoded by *Scn2a*, to Na_*V*_1.6, encoded by *Scn8a* at the AIS throughout development (Boiko et al., 2001, 2003). At the AIS, isoform specific biophysics of Na_*V*_1.2 and Na_*V*_1.6 can shape threshold, waveform, and compartment specific excitability in a developmentally regulated manner (Hu et al., 2009; Nelson and Jenkins, 2017). In the MNTB, Na_*V*_1.6 expression in the soma of principal neurons is thought to decrease over development but Na_*V*_1.6 remains abnormally expressed at later stages in deaf mice (Leao et al., 2006), indicating Na^+^ current composition is highly activity-dependent during development. However, there is no clear evidence Na_*V*_1.2 channel expression in MNTB neurons, specifically depending on auditory experience in activity-dependent manner before and after hearing onset. Here we demonstrated Na_*V*_ channel currents in MNTB principal neurons before and after hearing onset using *Scn2a* heterozygous mice.

Like many auditory nuclei, the MNTB is organized along a medial–lateral tonotopic axis, with systematic variation in cellular properties aligned to frequency tuning. In the MNTB, high-frequency responding neurons are located in the medial side, while low-frequency responding neurons are on the lateral side thus supporting distinct timing and firing demands (Sommer et al., 1993; Leao et al., 2006; Borst and Soria van Hoeve, 2012). A prominent organizing principle is that ion channel expression and conductance magnitudes vary across this axis. Previous studies demonstrated graded differences in K^+^ current amplitudes across the MNTB tonotopic map, implying that intrinsic membrane repolarization and temporal precision are tuned in a location-dependent manner. Consistent with this, voltage-gated potassium channel, K_*V*_3-family channels (e.g., K_*V*_3.1 and K_*V*_3.3), which are key determinants of fast spiking and narrow APs in auditory brainstem neurons, show tonotopic regulation with higher K_*V*_3.1 and K_*V*_3.3 expression in the medial/high-frequency end (Brew and Forsythe, 1995; Li et al., 2001; von Hehn et al., 2004; Leão et al., 2005; Strumbos et al., 2010). K_*V*_1.3 and K_*V*_2.2 are higher in the lateral/low-frequency end (Johnston et al., 2008; Gazula et al., 2010). Beyond conductance gradients, structural determinants of excitability also follow tonotopy. The AIS, a primary site for spike initiation, exhibits tonotopic differences in length and/or position in MNTB neurons and can undergo experience-dependent remodeling, linking circuit activity to intrinsic spike-generation machinery (Kim et al., 2019). Based on these findings, we tested whether *Scn2a* follows a tonotopic expression pattern and how Na_*V*_1.2 perturbation impacts auditory brainstem development.

In this study, we investigated how *Scn2a* haploinsufficiency shapes intrinsic Na^+^ channel function and spike output in MNTB principal neurons across development. We focused on two developmental stages: pre-hearing (P4–P11) and post-hearing onset (P14–P24). By combining whole-cell voltage-clamp measurements of Na^+^ currents with current-clamp recordings of APs, we found a pronounced pre-hearing reduction in Na^+^ current components in lateral MNTB neurons and genotype-dependent changes in AP kinetics across the tonotopic axis. These results suggest that Na_*V*_1.2 shapes early spike fidelity and waveform maturation and its tonotopic expression is important during MNTB development prior to hearing onset.

## 2 Materials and Methods

### 2.1 Animals

Animal studies were performed according to the regulations of the institutional animal care and use committee. Approval was obtained from the University of Michigan Institutional Animal Care and Use Committee (IACUC) under protocol PRO00012821 and in accordance with the National Institutes of Health’s Guide for the Care and Use of Laboratory Animals. All animals used in this study were under 12 light/12 dark cycle with *ad libitum* feeding and drinking. Conventional *Scn2a* mutant mice [*Scn2a*^+/+^ and *Scn2a*^+/–^ mice, C57BL6/J (RRID:IMSR JAX:000664)] background, were obtained from Kevin Bender (UCSF). Both sexes of control (*Scn2a*^+/+^) and *Scn2a*^+/–^ mice were used for all experiments performed in this study. All experiments were done between postnatal day 4–11 (P4-P11) and P14-24.

### 2.2 Brain slice preparation

After rapid decapitation of the mice, which were deeply anesthetized by isoflurane inhalation, the brainstem was quickly removed from the skull and immersed in ice-cold low-calcium artificial CSF (aCSF) containing the following (in mM): 125 NaCl, 2.5 KCl, 3 MgCl_2_, 0.1 CaCl_2_, 25 glucose, 25 NaHCO_3_, 1.25 NaH_2_PO_4_, 0.4 ascorbic acid, 3 myoinositol, and 2 Na-pyruvate, pH 7.3–7.4 when bubbled with carbogen (95% O_2_/5% CO_2_); osmolarity 310–320 mOsm/L. Transverse brainstem slices containing the MNTB were sectioned to 200 μM thickness using a Vibratome (Leica, VT1200S). Brainstem slices were then transferred to an incubation chamber containing normal aCSF bubbled with carbogen, where they were maintained for 30 min at 34–35°C and thereafter at room temperature (24°C). Normal aCSF was the same as low calcium aCSF, but with 1 mM MgCl_2_ and 2 mM CaCl_2_.

### 2.3 Electrophysiological recordings

Brainstem slices were perfused with normal aCSF at 2 ml/min and visualized using an infrared differential interference contrast microscope (Axio Examiner, Zeiss, Oberkochen, Germany) with a 63 × water-immersion objective and a CMOS camera (ORCA-Flash2.8, Hamamatsu, Japan). Whole-cell patch-clamp recordings were performed in normal aCSF at room temperature (24°C) using an EPC-10 amplifier controlled by PATCHMASTER software (HEKA, Elektronik, Lambrecht/Pfalz, Germany, RRID:SCR_000034). For recordings of Na^+^ currents, the pipettes were filled with a solution containing the following (in mM): 130 Cs-Methanesulfonate, 10 CsCl, 5 EGTA, 4 ATP-Mg, 10 HEPES, 0.3 GTP-Na, 5 Na_2_-phosphocreatine, 10 TEA-Cl, pH adjusted to 7.4 with CsOH and osmolarity of ∼290 mOsm/L. To isolate Na^+^ currents the following drugs were added to the bath recording solution to block K^+^ and Ca^2+^ channels (in mM): 10 TEA, 2 4-AP, 0.2 CdCl_2_. Glass borosilicate pipettes were pulled at 2.5–6 MΩ with glass puller (Sutter Instrument, P1000, Novato, CA, USA, RRID:SCR_021042) and coated with wax. Recordings were included in the dataset if R-series did not exceed 15 MΩ and compensated > 50% with a leak current > 200 pA. Liquid junction potential was 4.8 mV and recordings were not compensated. The holding potential was −70 mV in voltage-clamp mode. Sodium currents were recorded in voltage clamp protocols for 100 ms duration with voltage steps from −80 mV to +40 mV (10 mV increments). Signals were filtered at 10 kHz and sampled at 50 kHz. Internal pipettes included either Alexa 488 (Invitrogen, 1:500) or Biocytin (Invitrogen, 0.5%) to determine location along MNTB tonotopic axis. For recordings of MNTB principal neuron intrinsic properties, internal solution contained (in mM): 125 K-Gluconate, 20 KCl, 0.2 EGTA, 4 ATP-Mg, 10 HEPES, 0.3 GTP-Na, 5 Na_2_-phosphocreatine, pH 7.4 and osmolarity of ∼290 mOsm/L was used. To block low-voltage potassium channels 1 mM TEA-Cl was added to the bath recording solution. Similar parameters were used for recordings as stated in the previous section. Signals were filtered at 2.9 kHz and acquired at a sampling rate of 10–50 μs. Action potential (AP) excitability was recorded in current clamp for 200 ms duration with current steps from −100 pA to +400 pA in 50 pA increments. AP waveform parameters were analyzed from the first AP induced by minimum current injection (rheobase current) and the subsequent AP phase plot, where membrane potential slope (dV/dt) is plotted against the membrane potential. Thresholds were determined when dV/dt exceeds 10 V/s in the phase plot, amplitude as the max width in the phase plot (Wollet and Kim, 2022), maximum dV/dt (depolarization phase) as the peak in the phase plot, *y*-axis, and minimum dV/dt (depolarization phase) as the negative peak in the phase plot, *y*-axis. Cells that exceeded 18 action potentials were excluded in this dataset. Electrophysiological recordings were analyzed and displayed with Igor Pro (WaveMetrics, Lake Oswego, OR, United States) and AxoGraph (AxoGraph Scientific, Sydney, Australia).

### 2.4 Immunohistochemistry

Mouse brain slices were fixed with 4% paraformaldehyde in 0.1 M PBS for 10 min, or 8 min for Na^+^ staining, followed by 5 min washes in 0.1 M PBST [0.3% (w/v) Triton X-100)] three times. Free-floating slices were placed in 0.1 M PBST containing 5% BSA with primary antibody overnight at room temperature. Negative controls did not contain primary antibody. Primary antibodies used: anti-MAP2 (Mouse IgG1, Millipore, Cat#MAB3418, 1:500, RRID:AB_94856) or anti-MAP2 (Rabbit IgG, Millipore, Cat#AB5622, 1:250, RRID:AB_91929), anti-Na_*V*_1.2 (Mouse IgG2a, Neuromab, Cat#75-024, 1:100) and anti-Na_*V*_Pan (Mouse IgG1, Millipore, Cat#S8809, 1:500). Slices were washed in 0.1 M PBST three times for 5 min then incubated with corresponding secondary antibodies for 2 h at room temperature. Secondary antibodies used (Invitrogen): goat anti-mouse IgG or goat anti-rabbit IgG Alexa Fluor 647, goat anti-mouse IgG (anti-mouse IgG2a for Na_*V*_1.2) Alexa Fluor 488 and Streptavidin-conjugated 488 all at 1:500 dilution. Sections were washed three times, 5 min each, before mounted on microscope slides and cover slipped using Fluoromount-G (Invitrogen, E143217) mounting medium and sealed with clear nail polish. Stained slices were viewed at 488 nm, 568 nm, and 647 nm using a 20x/1.40, 40x/1.40 or 63x/1.40 oil-immersion objective on a confocal laser-scanning microscope (Nikon A1R, Tokyo, Japan, RRID:SCR_020317). Stack images were acquired at a digital size of 1024 × 1024 pixels with optical section separation (*z*-interval) of 0.5 μm and were later crop to the relevant part of the field without changing the resolution. The confocal image stacks were analyzed using ImageJ software (Fiji, RRID:SCR_002285). Length and distance of Na_*V*_Pan were measured using the segmented line tool in ImageJ software (Fiji). Principal neuron tonotopy in the MNTB was distinguished by the distance from midline. Medial neurons are in the most medial 30% of the MNTB and lateral neurons are in the most lateral 30% of the MNTB from midline.

### 2.5 Fluorescent *in situ* hybridization (FISH)

To examine the expression of voltage-gated sodium channel 1.2 and 1.6 (Na_*V*_ 1.2 and Na_*V*_ 1.6), Fluorescent *in situ* hybridization (FISH) was used to quantify transcription of *Scn2a* and *Scn8a* for Na_*V*_ 1.2 and Na_*V*_ 1.6, respectively. Brains from P4-5 and P19-20 were extracted and freshly frozen by submerging in 2-methylbutane chilled on dry ice. Brains were sectioned with 15–20 um thickness using cryostat, and the brain slices were directly mounted on Superfrost Plus microscope slides and were stored at −80°C until ready for use. Transcripts of *Scn2a* (ACD, Cat# 423641-C3) and *Scn8a* (ACD, Cat# 434191-C2) were labeled with respective probes using RNAscope™ Multiplex Fluorescent Reagent Kit v2 (ACD, Cat# 323100) by following manufacturer’s protocol. After FISH, the slices were stained with anti-MAP2 (Mouse IgG1, Millipore, Cat#MAB3418, 1:500, RRID:AB_94856) to visualize MNTB neurons on a confocal laser-scanning microscope (Nikon A1R, Tokyo, Japan, RRID:SCR_020317). To minimize variation between images, all the images were taken with the same laser power setting and pinhole size. Expression of *Scn2a* and *Scn8a* were quantified by measuring positive area of each signal within a cell by setting the same threshold in the Image J software (Fiji).

### 2.6 Statistical analysis

GraphPad Prism 9 & 10 Software (San Diego, CA, USA) was used for statistical analysis and displayed graphs. The Kolmogorov-Smirnov test was used for normality of datasets. Parametric or non-parametric tests were carried out according to results of the normality test. For two group comparisons, an Unpaired *t*-test with Welch’s correction or Mann-Whitney *U* test was performed. To measure the response to two factors, a two-way ANOVA with repeated measures was used, followed by Šídák’s *post hoc* comparisons or multiple comparisons with Bonferroni correction. The Geisser and Greenhouse correction was applied to correct violations of the assumption of sphericity. Data are represented in the text and figures mean ± SEM. Significant differences were defined as *p* < 0.05.

## 3 Results

### 3.1 Developmental reduction of Na_*V*_1.2-dependent Na^+^ currents in *Scn2a^+/–^* MNTB neurons during pre-hearing period with normalization after hearing onset

To investigate how *Scn2a* haploinsufficiency shapes intrinsic Na^+^ channel function and spike output in MNTB principal neurons across development, we examined whether Na_*V*_1.2 is present at MNTB principal neurons and whether its expression is regulated in a sound-evoked, activity-dependent manner across development. We first attempted to visualize Na_*V*_1.2 channel in the MNTB using immunohistochemistry. Na_*V*_1.2 -specific immunoreactivity was not reliably detected in MNTB neurons under our staining conditions (Supplementary Figure 1). This result likely reflects technical limitations that are well recognized for isoform-specific Na_*V*_1.2 antibodies, including low antigen abundance, epitope masking, sensitivity to fixation/permeabilization, and potential compartmental restriction to small axonal domains that are difficult to resolve by conventional immunostaining. In contrast, AIS structures were readily identifiable by robust pan-voltage-gated sodium channel (Na_*V*_Pan) labeling. Quantification of AIS geometry based on Na_*V*_Pan labeling (AIS length and position relative to the soma) revealed no significance difference between *Scn2a^+/+^* and *Scn2a^+/–^* mice (Supplementary Figure 1). Furthermore, fluorescent *in situ* hybridization (FISH) was performed during the pre-hearing (P4-5) and post-hearing (P19-20) stage to compare *Scn2a* (encoding Na_*V*_1.2) and *Scn8a* (encoding Na_*V*_1.6) expression in the MNTB (Supplementary Figure 2). *Scn2a* expression was higher during the pre-hearing stage, whereas *Scn8a* increased after hearing onset. The *Scn2a/Scn8a* ratio was significantly greater at P4–5 than at P19–20, indicating that Na_*V*_1.2 is enriched early in MNTB development and undergoes a developmental shift relative to Na_*V*_1.6.

Therefore, we pursued a complementary functional approach to test whether *Scn2a*, encoding Na_*V*_1.2, contributes to Na^+^ channel currents in developing MNTB neurons, using whole-cell voltage-clamp recordings. Because homozygous *Scn2a* loss is perinatal lethal, we used *Scn2a^+/–^* mice to assess the functional contribution of *Scn2a* to intrinsic Na^+^ currents during MNTB development. We quantified somatic Na^+^ currents (I_*Na*_) in MNTB principal neurons across development, focusing on pre-hearing (P4–P6) and post-hearing onset (P14–P19) periods. Previous studies determined that Na^+^ channel subtype contributions are dynamically regulated across postnatal maturation and can shift in a region and circuit-dependent manner, including developmental redistribution among Na_*V*_ channel isoforms in the CNS (e.g., Na_*V*_1.2 to Na_*V*_1.6) (Boiko et al., 2001, 2003) and reported changes in auditory brainstem pathways (Leao et al., 2006).

**FIGURE 1:**
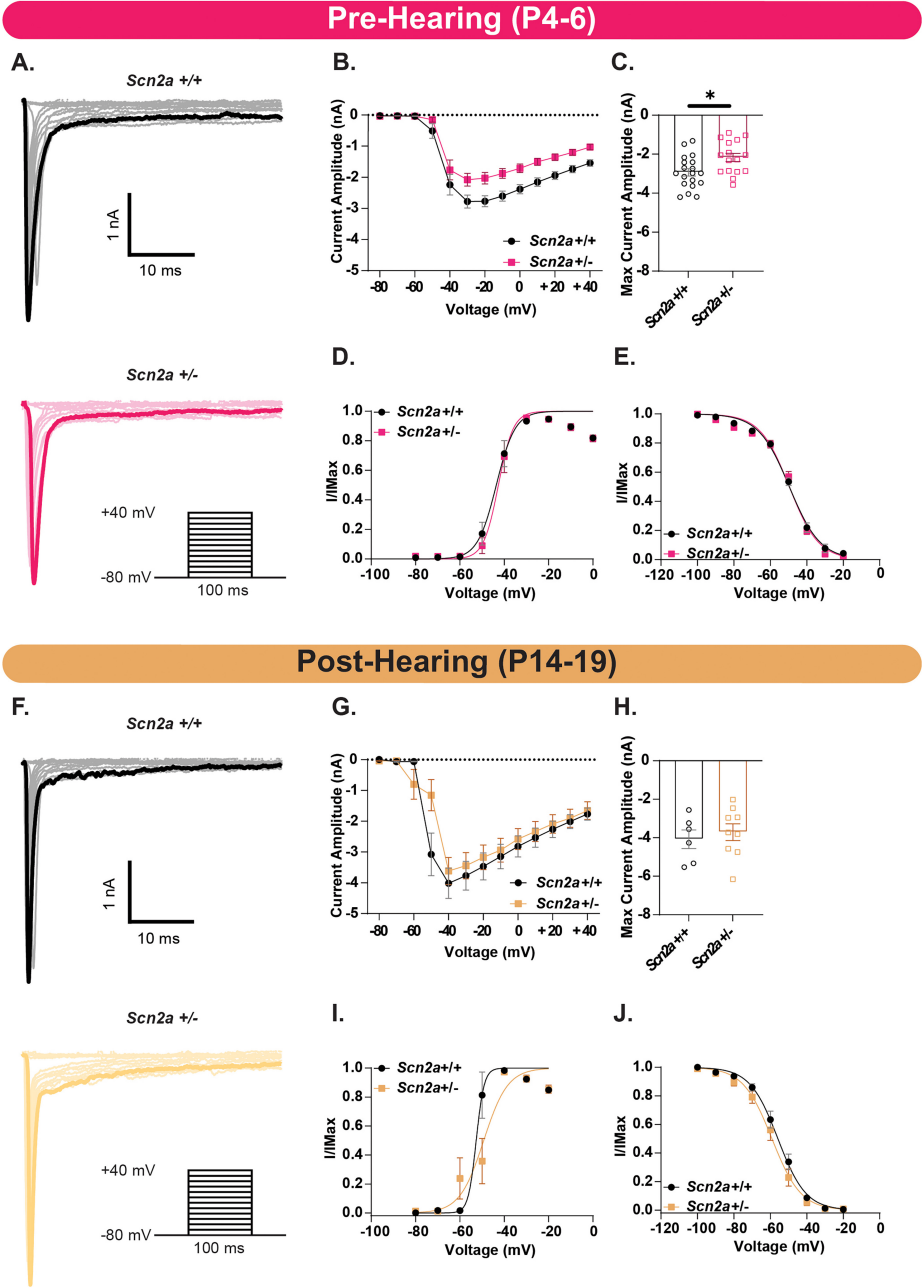
Voltage-gated sodium current was reduced in pre-hearing period and recovered after hearing onset. **(A–E)** Pre-hearing stage (P4-P6). **(A)** Representative whole-cell voltage-clamp recordings of transient inward Na^+^ currents (I_*Na*_) evoked by depolarizing steps. Inset, protocol schematic; 100 ms steps –80 to +40 mV. Light traces indicate responses across voltage steps; bold trace highlights a representative response with max currents. **(B)** Current-voltage (I-V) relationship curves for MNTB principal neurons from *Scn2a^+/+^* (black, *n* = 19 cells; *N* = 4 mice) and *Scn2a^+/–^* (pink, *n* = 16 cells; *N* = 4 mice). **(C)** Summary of maximal I_*Na*_ amplitude, which is reduced in *Scn2a^+/–^* relative to *Scn2a^+/+^*. Individual cells are plotted as open symbols. **(D)** Steady-state activation curve, which was obtained by normalization to I_max_, plotted, and fit with a Boltzmann function. **(E)** Steady-state inactivation curve (normalized to I_max_) fit with a Boltzmann function for MNTB neurons from *Scn2a^+/+^* (black) and *Scn2a^+/–^* mice (pink). **(F–J)** Post-hearing stage (P14–P19). **(F)** Representative I_*Na*_ traces recorded with the same voltage-step protocol. **(G)** I–V relationship for MNTB neurons from *Scn2a^+/+^* (black) and *Scn2a^+/–^* mice (orange). **(H)** Summary of maximal I_*Na*_ amplitude showing no significant genotype difference at P14–P19. **(I)** Steady-state activation and **(J)** steady-state inactivation curves (normalized; Boltzmann fits) comparing genotypes after hearing onset. Data are presented as mean ± SEM. *p < 0.05, Unpaired *t*-test.

Using whole-cell voltage-clamp recordings during the pre-hearing stage (P4–P6, both sexes), we observed a reduction in transient I_*Na*_ in *Scn2a^+/–^* MNTB principal neurons compared with *Scn2a^+/+^* littermates across the current–voltage (I–V) relationship (Figures 1A,B). Consistent with this, the maximal inward current was significantly smaller in *Scn2a^+/–^* neurons (Unpaired *t*-test: *Scn2a^+/+^*, −2.94 ± 0.19 nA vs. *Scn2a^+/–^*, −2.17 ± 0.21 nA; *t* = 2.7, *p* = 0.0108; *n* = 19 and 16 cells, respectively; Figure 1C). In contrast, steady-state gating properties were largely preserved at this age. Boltzmann fits of the activation curves yielded identical half-activation voltages (V_1/2_ = −43 mV for both genotypes) with similar slope factors (*Scn2a^+/+^*, *k* = 4.24; *Scn2a^+/–^*, *k* = 3.34; Figure 1D and Table 1). Likewise, steady-state inactivation parameters were comparable (*Scn2a^+/+^*: V_1/2_ = −49.58 mV, *k* = −8.46; *Scn2a^+/–^*: V_1/2_ = −49.6 mV, *k* = −8; Figure 1E and Table 1). These data indicate that loss of one *Scn2a* allele primarily reduces I_*Na*_ during early postnatal development without producing major changes in Na^+^ channel voltage- dependence or kinetics.

**TABLE 1:** Pre-hearing and post-hearing onset sodium channel kinetic properties.

	Pre-hearing onset (P4-6)		Post-hearing onset (P14-19)	
Values represented as Mean ± SEM	*Scn2a^+/+^*	*Scn2a^+/–^*	*Scn2a^+/+^*	*Scn2a^+/–^*
Max sodium current (nA)	−2.94 ± 0.19^A^	−2.17 ± 0.21^B^	−4.08 ± 0.48^A^	−3.71 ± 0.43^A^
Activation curve – V_1/2_ (mV)	−43.58 ± 0.18^A^	−42.63 ± 0.18^A^	−52.65 ± 0.15^A^	−49.26 ± 0.25^A^
Activation curve - slope (*k*)	4.24 ± 0.18^A^	3.34 ± 0.18^A^	1.8 ± 0.15^A^	5.42 ± 0.25^A^
Inactivation curve – V_1/2_ (mV)	−49.58 ± 0.09^A^	−49.6 ± 0.08^A^	−55.85 ± 0.07^A^	−59.12 ± 0.08^B^
Inactivation curve - slope (*k*)	−8.46 ± 0.09^A^	−8 ± 0.08^A^	−7.68 ± 007^A^	−7.94 ± 0.08^A^

Means are calculated within grouped columns (i.e., *Scn2a^+/+^* or *Scn2a^+/–^*). Within a row, means superscripted with A are statistically different from means superscripted with B.

Furthermore, a subset of immature *Scn2a^+/+^* MNTB principal neurons exhibited a time-delayed inward Na^+^ current component (9/19 cells; ∼53%) with a mean amplitude of 2.04 ± 0.33 nA (*N* = 4 mice; Supplementary Figure 3). In contrast, none of *Scn2a^+/–^* MNTB principal neurons displayed this time-delayed Na^+^ current component (0/16 cells; *N* = 5 mice). Delayed-onset Na^+^ currents that appears to “backpropagate” into the somatic recording as the axonal compartments such as the AIS and/or nearby axonal nodes are recruited (Hu et al., 2009). These delayed-onset Na^+^ currents recorded under somatic voltage clamp are consistent with recruitment of electrotonically remote axonal Na_*V*_ channels. The resulting current is detected at the soma due to disrupted somatic clamp commands (Barlow et al., 2024) and can therefore appear with a latency as the axonal compartment becomes activated (Kole et al., 2008; Baranauskas et al., 2013). Nevertheless, the complete loss of this component in *Scn2a^+/–^* neurons together with the reduction in peak somatic I_*Na*_ amplitude provides convergent functional evidence that *Scn2a*/Na_*V*_1.2 contributes to Na^+^ channel activity in developing MNTB principal neurons during the pre-hearing stage.

We next examined whether this reduction persists after hearing onset, which occurs around P12–P14 in mice (Saunders et al., 1980). At P14–P19, peak I_*Na*_ amplitudes in *Scn2a^+/–^* neurons were comparable to controls across the I–V relationship (Figures 1F,G), and maximal inward current amplitude was not detectably different between genotypes (Figure 1H). Steady-state activation remained similar following hearing onset, with Boltzmann fits showing *Scn2a^+/+^*, V_1/2_ = −52.65 mV and *k* = 1.8, and *Scn2a^+/–^*, V_1/2_ = −49.26 mV and *k* = 5.42 (Figure 1I and Table 1). Steady-state inactivation curves were also closely matched between genotypes (*Scn2a^+/+^*, V_1/2_ = −55.85 mV, *k* = −7.68; *Scn2a^+/–^*, V_1/2_ = −59.12 mV, *k* = −7.94; Figure 1J and Table 1). Thus, while *Scn2a* haploinsufficiency produces a significant reduction in transient I_*Na*_ amplitude during the pre-hearing period, peak I_*Na*_ magnitude and overall voltage-dependent gating are largely preserved after hearing onset, consistent with a developmentally restricted contribution of Na_*V*_1.2 to total Na^+^ conductance in MNTB principal neurons.

### 3.2 Tonotopic gradient of Na_*V*_1.2-dependent transient and persistent Na^+^ currents in *Scn2a^+/–^* MNTB neurons during the pre-hearing period

The MNTB is organized along a medial–lateral tonotopic axis, with medial principal neurons associated with higher-frequency inputs and lateral neurons associated with lower-frequency inputs (Weatherstone et al., 2017; Wollet and Kim, 2022). Voltage gated potassium channels such as K_*V*_3.1 or K_*V*_1.2 are differentially expressed along tonotopic axis of the MNTB during postnatal development (Wang et al., 1998; Dodson et al., 2002; Dodson et al., 2003; Ishikawa et al., 2003; von Hehn et al., 2004). Because tonotopic specialization can be accompanied by molecular and physiological gradients, we asked whether *Scn2a* haploinsufficiency produces region-specific effects on Na^+^ channel function along this tonotopic axis in the MNTB.

**FIGURE 2:**
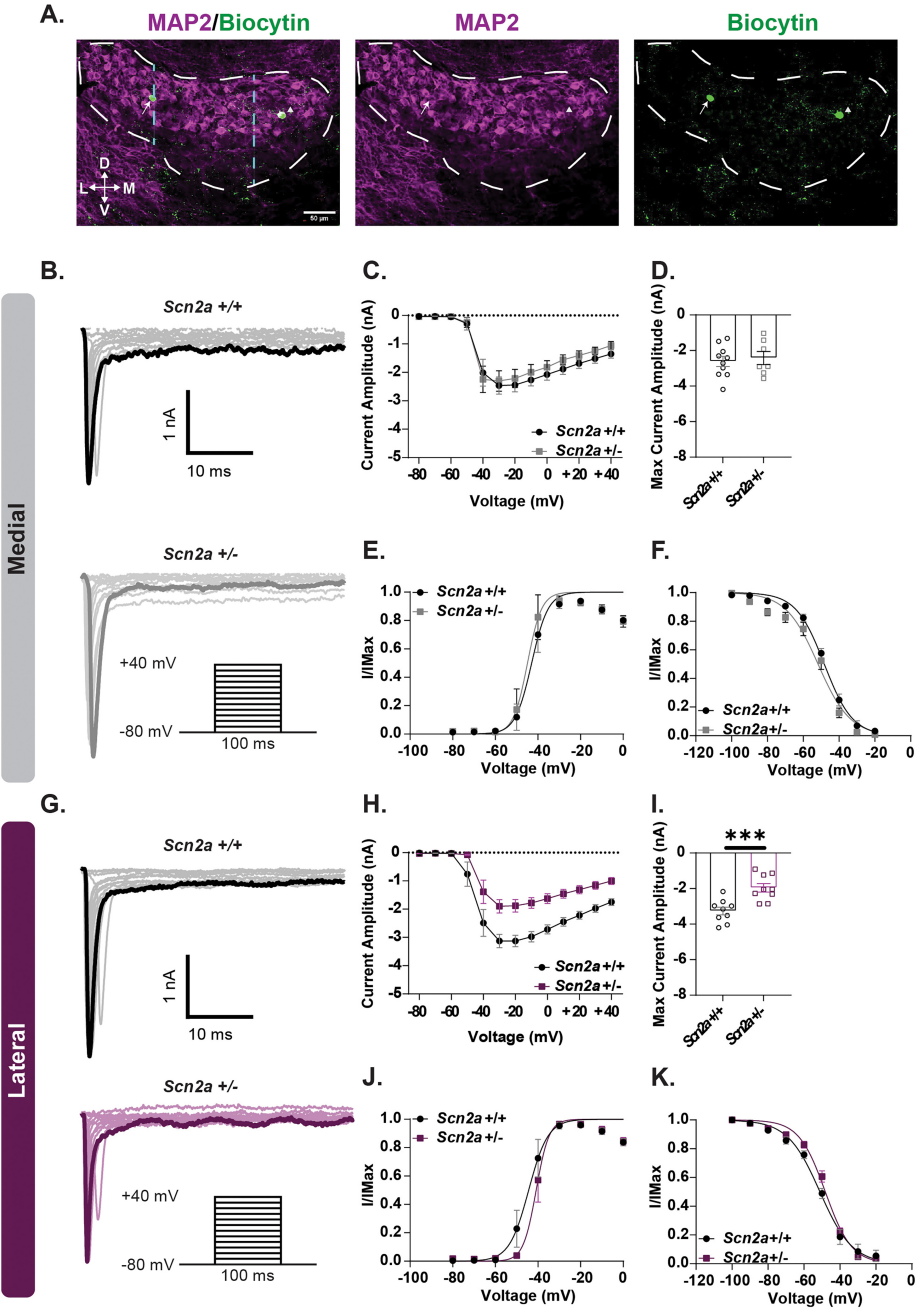
Regional differences in neuronal activation and voltage-gated Na^+^ currents in *Scn2a* haploinsufficient MNTB neurons during early development. **(A)** Immunofluorescence images of the mouse MNTB, showing MAP2 (magenta) labeling MNTB principal neurons along tonotopic axis of the MNTB, delineated by the white dashed outline. During whole-cell recordings, MNTB neurons are marked with Biocytin (green). Arrow indicates lateral MNTB neuron and arrowhead indicates medial MNTB neuron. Vertical blue dashed lines represent thresholds for lateral and medial location of MNTB neurons (<30% along MNTB length from medial edge). Orientation axes are shown (D, dorsal; V, ventral; M, medial; L, lateral). Scale bar, 50 μm. **(B–F)** Medial MNTB neuron recordings. **(B)** Representative whole-cell voltage-clamp recordings of transient I_*Na*_ evoked by depolarizing voltage steps in *Scn2a^+/+^* (top, black) and *Scn2a^+/–^* mice (bottom, gray) MNTB neurons. Light traces indicate responses across the voltage-step series; bold trace highlights a representative response with maximal amplitude. The voltage-step protocol is depicted (100 ms steps from approximately –80 to +40 mV). **(C)** I–V relationship for medial MNTB neurons in *Scn2a^+/+^* (*n* = 10 cells; *N* = 3 mice) and *Scn2a^+/–^* (*n* = 7 cells; *N* = 3 mice). **(D)** Summary of maximal I_*Na*_ amplitude for medial MNTB neurons; individual cells are plotted. Steady-state activation **(E)** and inactivation **(F)** curves (normalized, Boltzmann fits shown) comparing genotypes in medial MNTB neurons. **(G–K)** Lateral MNTB neurons recordings. **(G)** Representative I_*Na*_ recorded from lateral MNTB neurons in *Scn2a^+/+^* (top, black) and *Scn2a^+/–^* mice (bottom, purple). **(H)** I–V relationship for lateral MNTB neurons. **(I)** Summary of maximal I_*Na*_ amplitude in lateral MNTB neurons. Steady-state activation **(J)** and inactivation **(K)** curves (normalized, Boltzmann fits) for lateral MNTB neurons. Data are presented as mean ± SEM. ***p < 0.001, Unpaired *t*-test.

Using whole-cell voltage-clamp recordings from anatomically defined medial and lateral MNTB principal neurons [Figure 2A; see also (Kim et al., 2019)], we quantified transient I_*Na*_ and their voltage-dependent gating during the pre-hearing stage. In medial MNTB principal neurons, transient I_*Na*_ amplitudes were comparable between genotypes, indicating peak current magnitude was preserved (Figures 2B–D). Steady-state activation and inactivation were unchanged in medial MNTB neurons (Figures 2E,F and Table 2). Together, these data indicate that medial MNTB neurons are relatively spared with respect to I_*Na*_ amplitude.

**TABLE 2:** Medial nucleus of the trapezoid body (MNTB) tonotopic axis sodium channel kinetic properties.

	Medial		Lateral	
Values represented as Mean ± SEM	*Scn2a^+/+^*	*Scn2a^+/–^*	*Scn2a^+/+^*	*Scn2a^+/–^*
Max sodium current (nA)	−2.61 ± 0.28^A^	−2.43 ± 0.37^A^	−3.27 ± 0.64^A^	−1.97 ± 0.72^B^
Activation Curve – V_1/2_ (mV)	−42.94 ± 0.18^A^	−44.91 ± 0.19^A^	−44.38 ± 0.19^A^	−40.86 ± 0.16^B^
Activation curve - slope (*k*)	3.86 ± 0.18^A^	3.32 ± 0.19^A^	4.55 ± 0.19^A^	3.04 ± 0.16^A^
Inactivation curve – V_1/2_ (mV)	−48.24 ± 0.08^A^	−51.74 ± 0.1^B^	−51.13 ± 0.1^A^	−48.14 ± 0.07^B^
Inactivation curve - slope (*k*)	−7.9 ± 0.08^A^	−8.99 ± 0.1^A^	−8.99 ± 0.1^A^	−7.33 ± 0.07^A^

Means are calculated within grouped columns (i.e., *Scn2a^+/+^* or *Scn2a^+/–^*). Within a row, means superscripted with A are statistically different from means superscripted with B.

In lateral MNTB principal neurons, *Scn2a* haploinsufficiency produced a robust reduction in transient I_*Na*_ amplitude (Figures 2G–I). Maximal inward current was significantly decreased in *Scn2a*^+/–^ neurons compared with *Scn2a^+/+^* controls (Unpaired *t*-test: *Scn2a^+/+^*, −3.27 ± 0.64 nA vs. *Scn2a*^+/–^, −1.97 ± 0.72 nA; *t* = 4.04, *p* = 0.001; *n* = 9 cells for both groups; Figure 2I). In addition, both activation and inactivation curves were slightly rightward shifted in *Scn2a*^+/–^ lateral neurons (Figures 2J,K and Table 2), consistent with altered voltage dependence of channel recruitment that could reduce availability at subthreshold membrane potentials. Activation curve for *Scn2a^+/+^* (V_1/2_ = −44.38 mV, *k* = 4.55) and *Scn2a*^+/–^ (V_1/2_ = −40.86 mV, *k* = 3.04) MNTB principal neurons. Inactivation curves for *Scn2a^+/+^* (V_1/2_ = −51.13, *k* = −8.99) and *Scn2a*^+/–^ (V_1/2_ = −48.14, *k* = −7.33) neurons, indicating a genotype-dependent change in voltage-dependent gating in the same population that shows reduced peak current amplitude. Thus, during early development, the functional impact of *Scn2a* haploinsufficiency is strongest in low-frequency responding, lateral MNTB neurons, revealing a clear tonotopic gradient in I_*Na*_ disruption.

**FIGURE 3:**
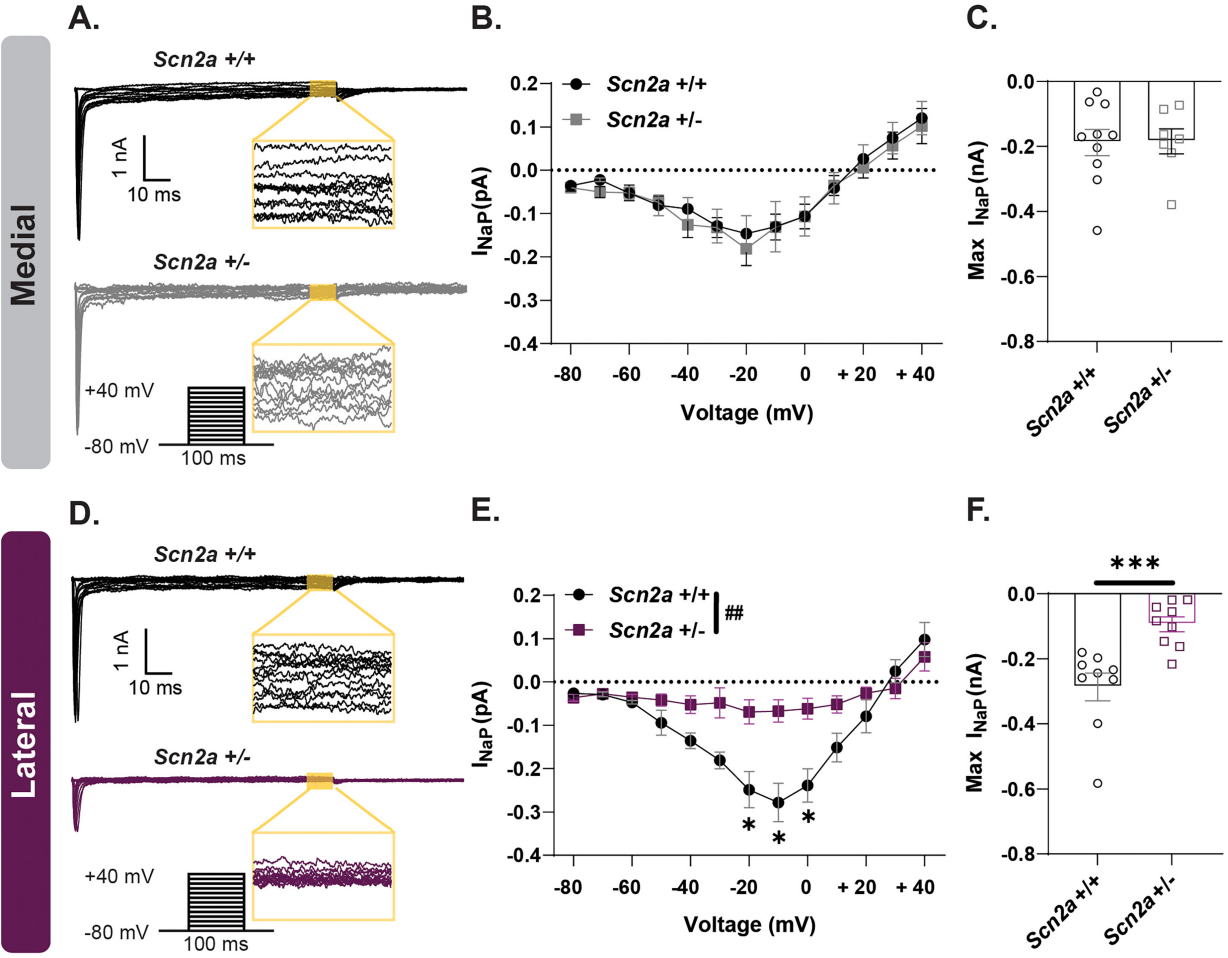
Regional-specific reduction of persistent Na^+^ current (I_*NaP*_) in lateral MNTB neurons of *Scn2a^+/–^* mice. **(A–C)** Medial MNTB neuron recordings. **(A)** Representative whole-cell voltage-clamp recordings illustrating the I_*NaP*_ in *Scn2a^+/+^* (top, black) and *Scn2a^+/–^* mice (bottom, gray) medial MNTB neurons evoked by depolarizing voltage steps. Insets (yellow box) show an expanded view of the late, non-inactivating current, I_*NaP*_, measured as the last 10 ms of the voltage step. **(B)** I–V relationship of I_*NaP*_ for medial MNTB neurons show no difference among medial MNTB principal neurons. **(C)** Summary of maximal I_*NaP*_ amplitude in medial MNTB neurons from *Scn2a^+/+^* (*n* = 10 cells; *N* = 3 mice) and *Scn2a^+/–^* (*n* = 7 cells; *N* = 3 mice). **(D–F)** Lateral MNTB neuron recordings. **(D)** Representative recordings from *Scn2a^+/+^* (top, black) and *Scn2a^+/–^* mice (bottom, purple), with insets highlighting the I_*NaP*_. Voltage protocol as in panel **(A)**. **(E)** I-V relationship curves of I_*NaP*_ for lateral MNTB neurons demonstrating reduced I_*NaP*_ in lateral *Scn2a^+/–^* neurons (*n* = 9 cells; *N* = 4 mice) compared to *Scn2a^+/+^* neurons (*n* = 9 cells; *N* = 3 mice). There is a significant difference between –20 to 0 mV. **(F)** Summary of maximal I_*NaP*_ amplitude in lateral MNTB neurons showing a significant reduction in *Scn2a^+/–^* relative to *Scn2a^+/+^* mice. Data are presented as mean ± SEM. ^##^*p* < 0.01, two-way ANOVA and *p < 0.05, Šídák’s multiple comparisons test for panel **(E)** and ***p < 0.001, Mann-Whitney *U* test for panel **(F)**.

Given the gating differences observed for transient currents (Figure 2), we next examined whether *Scn2a* haploinsufficiency also affects the persistent Na^+^ current (I_*NaP*_), which can shape subthreshold excitability and spike initiation. Strikingly, I_*NaP*_ showed a similar tonotopic pattern (Figure 3). In medial MNTB neurons, I–V relationships for I_*NaP*_ and maximal I_*NaP*_ amplitude did not differ by genotype (Figures 3A–C). In contrast, lateral MNTB neurons exhibited a significant reduction in I_*NaP*_ across the I–V relationship (two-way ANOVA: *Scn2a^+/+^*, −0.1069 nA vs. *Scn2a*^+/–^, −0.0364 nA; F(1,16) = 9.683, *p* = 0.0067; *n* = 9 cells for both groups; Figures 3D,E), with the most pronounced differences at depolarized subthreshold potentials (Šídák’s multiple comparisons: −20 mV, *Scn2a^+/+^*, −0.2486 ± 0.05 vs. *Scn2a*^+/–^, −0.069 ± 0.05 nA, *p* = 0.0384; −10 mV, *Scn2a^+/+^*, −0.278 ± 0.05 vs. *Scn2a*^+/–^, −0.067 ± 0.05 nA, *p* = 0.016; 0 mV, *Scn2a^+/+^*, −0.2389 ± 0.04 vs. *Scn2a*^+/–^, −0.0617 ± 0.04 nA, *p* = 0.0221; Figure 3E). Consistently, maximal I_*NaP*_ was also reduced in lateral *Scn2a*^+/–^ (Mann–Whitney *U* test: *Scn2a^+/+^*, −0.2865 ± 0.04 nA vs. *Scn2a*^+/–^, −0.0938 ± 0.02 nA; *U* = 2, *p* = 0.0002; Figure 3F). Notably, these tonotopic differences in I_*NaP*_ were not observed after hearing onset (Supplementary Figure 4), indicating that the reduction in I_*NaP*_ is developmentally restricted.

### 3.3 Na_*V*_1.2 reduction slows spike kinetics and broadens action potentials in developing MNTB principal neurons

**FIGURE 4:**
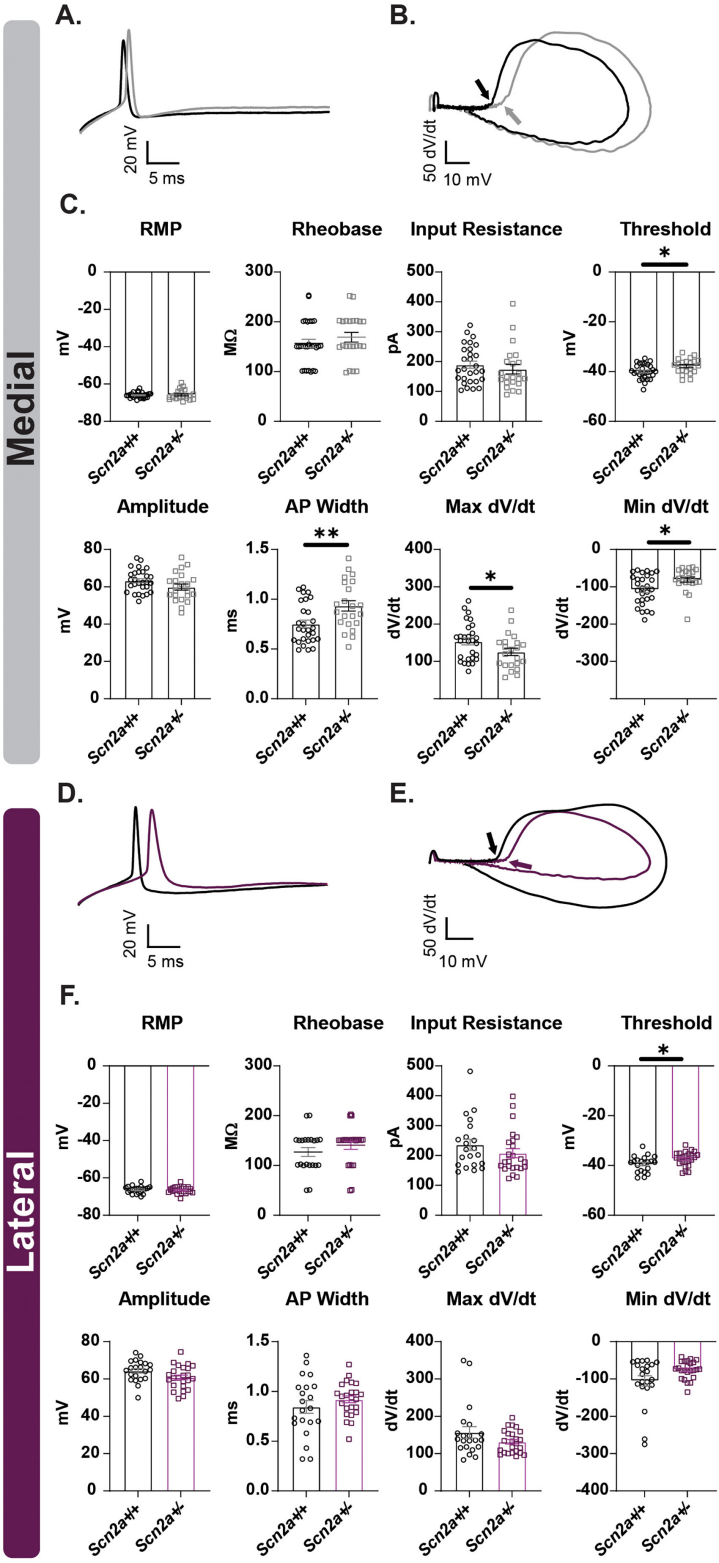
*Scn2a* haploinsufficiency alters intrinsic properties and action potential waveform of MNTB neurons in a region-dependent manner during the pre-hearing stage. **(A–C)** Medial MNTB principal neuron action potential waveform. **(A)** Representative single action potentials (APs) recorded in whole-cell current clamp from medial MNTB neurons of *Scn2a^+/+^* (black) and *Scn2a^+/–^* (gray) mice. **(B)** Representative phase plots (dV/dt vs. membrane potential) from medial MNTB neurons illustrating altered AP initiation and waveform kinetics in *Scn2a^+/–^* neurons (arrows indicate AP threshold during the rising phase). **(C)** Summary of intrinsic and AP waveform properties in medial MNTB neurons, including RMP, rheobase, input resistance, AP threshold, AP amplitude, AP width, maximal upstroke slope (max dV/dt), and maximal repolarization slope (min dV/dt). **(D–F)** Lateral MNTB principal neuron action potential waveform. **(D)** Representative AP waveforms recorded from lateral MNTB neurons in *Scn2a^+/+^* (black) and *Scn2a^+/–^* (purple) mice. **(E)** Representative phase-plane plots from lateral MNTB neurons. Arrows denote AP thresholds. **(F)** Quantification of intrinsic and AP waveform parameters in lateral MNTB neurons. Data are presented as mean ± SEM. *p < 0.05, Unpaired *t*-test (threshold and max dV/dt) and *p < 0.05, **p < 0.01, Mann-Whitney *U* test (min dV/dt and AP width).

We found the genotype-dependent reductions in transient and persistent I_*Na*_ during the pre-hearing period most prominently in lateral MNTB neurons (Figures 1–3). We next determined whether these biophysical deficits translate into altered action potential (AP) waveform properties in MNTB principal neurons. To address this, we performed whole-cell current-clamp recordings across development, comparing pre-hearing (P4–P11) and post-hearing (P14–P24) stages. APs were evoked at rheobase to standardize comparisons across cells, and waveform kinetics were further quantified using phase-plane analysis (dV/dt versus membrane potential) to sensitively capture changes in spike initiation and repolarization dynamics (Figure 4).

During the pre-hearing stage, *Scn2a^+/–^* medial MNTB principal neurons exhibited significant alterations in AP waveform consistent with slowed spike kinetics. Specifically, AP threshold was modestly depolarized in *Scn2a^+/–^* neurons compared with *Scn2a^+/+^* controls (Unpaired *t*-test: *Scn2a^+/+^*,−39.98 ± 0.57 mV vs. *Scn2a*^+/–^, −37.83 ± 0.64 mV; *t* = 2.525, *p* = 0.0149; Figure 4C). In parallel, the maximum upstroke velocity (max dV/dt), which is a sensitive readout of available Na^+^ conductance, was reduced (Unpaired *t*-test: *Scn2a^+/+^*, 153.6 ± 9.51 vs. *Scn2a*^+/–^, 125.6 ± 9.9 dV/dt; *t* = 2.016, *p* = 0.0494; Figure 4C), and the maximal repolarization rate (min dV/dt) was also decreased (Mann–Whitney *U* test: *Scn2a^+/+^*,−107.8 ± 7.62 vs. *Scn2a*^+/–^, −80.85 ± 6.68 dV/dt; *U* = 727, *p* = 0.0148; Figure 4C). Consistent with these kinetic changes, AP width was increased in *Scn2a*^+/–^ medial neurons (Mann–Whitney *U* test: *Scn2a^+/+^*, 0.7514 ± 0.04 vs. *Scn2a*^+/–^, 0.9341 ± 0.05 ms; *U* = 173.5, *p* = 0.0078; Figures 4A–C).

In lateral MNTB principal neurons, the effect on intrinsic waveform properties was more restricted. AP threshold was similarly depolarized in *Scn2a*^+/–^ neurons (Unpaired *t*-test: *Scn2a^+/+^*, −39.42 ± 0.7 mV vs. *Scn2a*^+/–^, −37.36 ± 0.6 mV; *t* = 2.262, *p* = 0.0288; Figures 4D–F), and AP width showed a trend toward broadening (*p* = 0.0537), while other waveform parameters were largely comparable between genotypes. Together, these findings indicate that reduced Na_*V*_1.2-dependent current during early development is accompanied by slower AP rise and repolarization and broader spikes in MNTB principal neurons.

Importantly, these AP waveform differences were not detected after hearing onset (Supplementary Figure 5), consistent with the recovery of peak Na^+^ current amplitude observed at P14–P19 (Figure 1) and supporting the conclusion that Na_*V*_1.2 plays a particularly important role in maintaining rapid and reliable spike kinetics before hearing onset. Notably, the magnitude of waveform disruption was greatest at P8–P11, aligning with the developmental period when Na^+^ channel composition and localization are thought to mature, including reported shifts in Na_*V*_ channel subtype contributions (Boiko et al., 2001, 2003; Leão et al., 2005). These data therefore place the functional impact of *Scn2a* haploinsufficiency within a defined early developmental window in the MNTB, motivating our subsequent analyses of how these intrinsic changes relate to sustained firing output and overall excitability.

### 3.4 MNTB principal neurons action potential excitability is not affected during development

Medial nucleus of the trapezoid body principal neuron excitability was preserved across development in *Scn2a* haploinsufficient mice. Given the reduction in I_*Na*_ amplitude and I_*NaP*_ during the pre-hearing period (Figures 1–3), we examined whether these early biophysical changes translate into altered spike output. Because *Scn2a* haploinsufficiency has been linked to neuronal hyperexcitability in other brain regions, including neocortex and cerebellum (Spratt et al., 2021; Wang et al., 2024), we hypothesized that MNTB principal neurons might exhibit increased firing during sustained depolarization, potentially reflecting compensatory mechanisms engaged in response to reduced Na_*V*_1.2-dependent conductance.

**FIGURE 5:**
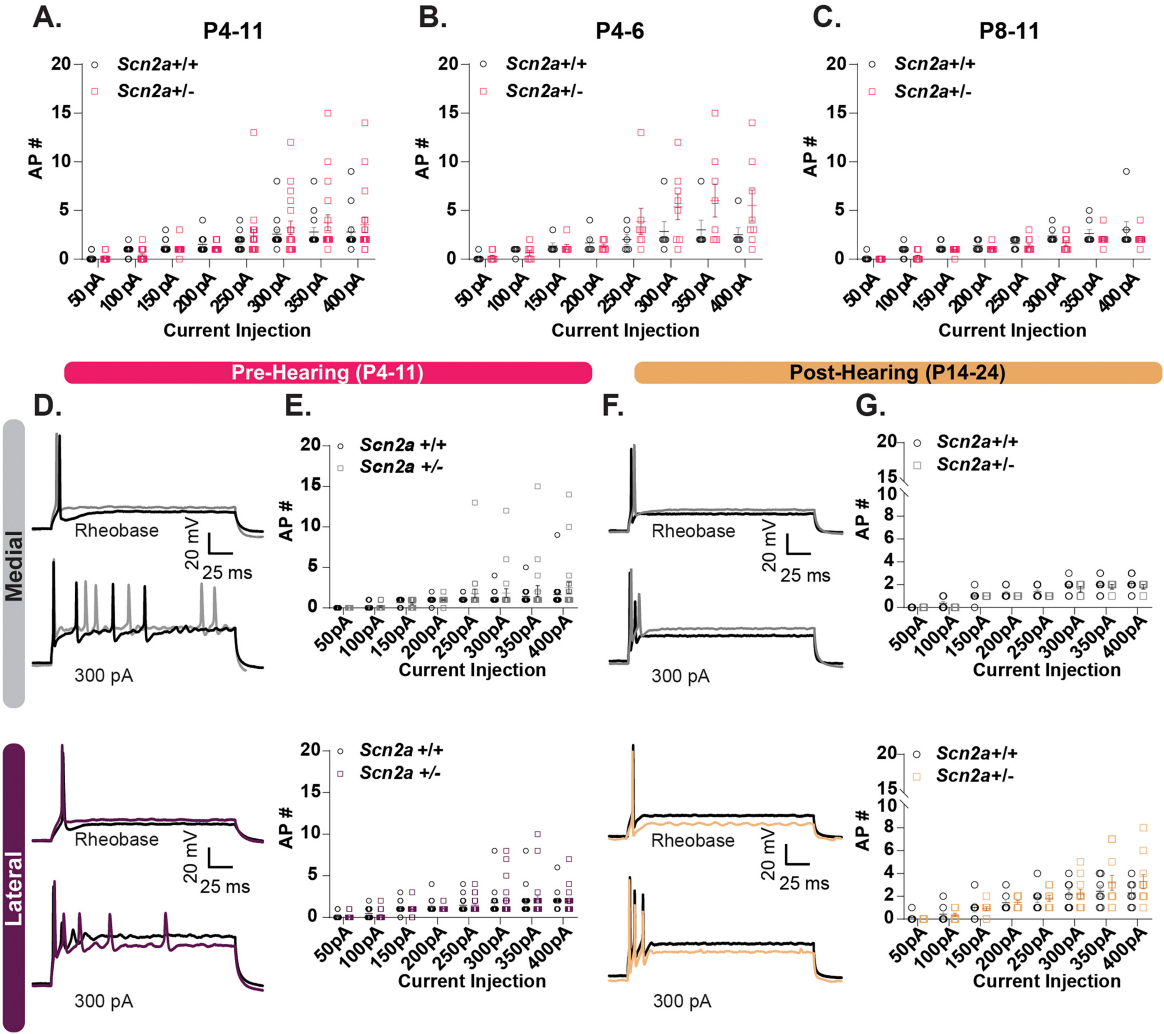
Medial nucleus of the trapezoid body (MNTB) principal neurons action potential excitability pre and post hearing onset. **(A)** Number of AP, during the pre-hearing stage (P4-P11), on a subset of MNTB neurons generating repetitive firing evoked across current injections (50–400 pA) in *Scn2a^+/+^* (black, *n* = 14cells, *N* = 8 mice) and *Scn2a^+/–^* (pink, *n* = 19 cells, *N* = 9 mice) MNTB neurons. **(B)** Increase trend of repetitive firing observed during the early stages of MNTB development (P4-P6) *Scn2a^+/+^* (*n* = 6 cells, *N* = 4 mice) compared to *Scn2a^+/–^* (*n* = 8 cells, *N* = 3 mice) but, abolished prior to hearing onset **(C)**, P8-P11 *Scn2a^+/+^* (*n* = 8 cells, *N* = 4 mice) compared to *Scn2a^+/–^* (*n* = 11 cells, *N* = 6 mice). **(D)** Representative whole-cell current-clamp traces from medial (top panel) and lateral MNTB neurons (bottom panel) in *Scn2a^+/+^* (black) and *Scn2a^+/–^* (gray and purple) mice in response to depolarizing current steps at rheobase and 300 pA, illustrating increase trend for spike output during sustained stimulation during the pre-hearing stage. **(E)** Frequency–current relationship expressed as the number of APs evoked during depolarizing current injections (50–400 pA) in medial (top panel) and lateral MNTB neurons (bottom panel). Individual neurons are plotted (open circles, *Scn2a^+/+^* vs. open squares, *Scn2a^+/–^*), showing no difference in AP number from 0 to 400 pA current steps in medial MNTB neurons (*Scn2a^+/+^*, *n* = 28 cells, *N* = 11 mice vs. *Scn2a^+/–^*, *n* = 22 cells, *N* = 9 mice) or lateral MNTB principal neurons (*Scn2a^+/+^*, *n* = 20 cells, *N* = 8 mice vs. *Scn2a^+/–^*, *n* = 24 cells, *N* = 9 mice. **(F)** Representative current-clamp responses from post-hearing (P14-P24) medial (top panel) and lateral (bottom panel) MNTB neurons in *Scn2a^+/+^* (black) and *Scn2a^+/–^* (gray and orange) mice during depolarizing current steps at rheobase and 300 pA. **(G)** Number of APs evoked across current injections (50–400 pA) in lateral MNTB neurons. AP numbers were similar in medial (*Scn2a^+/+^*, *n* = 11 cells, *N* = 5 mice vs. *Scn2a^+/–^*, *n* = 5 cells, *N* = 3 mice) and lateral MNTB principal neurons (*Scn2a^+/+^*, *n* = 7 cells, *N* = 5 mice vs. *Scn2a^+/–^*, *n* = 11 cells, *N* = 5 mice). Data are presented as mean ± SEM.

We performed whole-cell current-clamp recordings and evoked firing with a series of 200-ms depolarizing current steps (50–400 pA). As expected for this fast-spiking auditory brainstem population, most MNTB neurons fired one to two spikes near rheobase (typically ∼150 pA), whereas a subset of neurons generated repetitive firing at higher current injections (>300 pA) in both genotypes (Figure 5A). At the earliest pre-hearing stage (P4–P6), spike output between the genotypes was modestly increased in *Scn2a*^+/–^ neurons compared with *Scn2a^+/+^* controls, but did not have statistical significance (two-way ANOVA: *Scn2a^+/+^*, 1.98 AP vs. *Scn2a*^+/–^, 3.45 AP; F(1, 12) = 2.05, *p* = 0.177; Figure 5B). However, this trend was not maintained with maturation: by P8–P11, repetitive firing was reduced overall (Figures 5B,C).

Consistent with these age-stratified observations, across the full range of 0–400 pA current steps we did not detect genotype-dependent differences in the number of evoked action potentials in either medial MNTB neurons (*Scn2a^+/+^*, *n* = 28 cells, *N* = 11 mice; *Scn2a^+/–^*, *n* = 22 cells, *N* = 9 mice; Figures 5D,E, top panels) or lateral MNTB neurons (*Scn2a^+/+^*, *n* = 20 cells, *N* = 8 mice; *Scn2a^+/–^*, *n* = 24 cells, *N* = 9 mice; Figures 5D,E, bottom panels). Moreover, excitability remained comparable between genotypes after hearing onset, P14-P24 (Figures 5F,G). Together, these results indicate that although *Scn2a* haploinsufficiency reduces Na_*V*_1.2-dependent Na^+^ conductance and alters action potential waveform during the pre-hearing stage, these changes are not sufficient to measurably impact repetitive firing output in MNTB principal neurons before or after hearing onset.

## 4 Discussion

Our study identifies a developmentally restricted and tonotopically patterned role for Na_*V*_1.2 in shaping excitability of MNTB neurons. Before hearing onset, *Scn2a* haploinsufficiency results in reduced Na^+^ influx, with the most pronounced deficits in lateral MNTB neurons for both transient I_*Na*_ and the persistent component I_*NaP*_. After hearing onset, peak I_*Na*_ amplitude largely normalized, consistent with developmental reorganization of Na^+^ channel contributions. In parallel, current-clamp recordings revealed that *Scn2a* haploinsufficiency leads to slower and broader APs during the pre-hearing window, but repetitive firing output during prolonged current steps was preserved. These results indicate that *Scn2a* reduction impacts spike kinetics more strongly than spike output during sustained depolarization.

### 4.1 Compartment and conductance specific mechanisms dissociate somatic Na^+^ current amplitude from AP waveform changes

We observed that pronounced I_*Na*_ reduction occurred in lateral MNTB neurons, whereas AP waveform changes were detectable in both lateral and medial neurons (Figures 2–4). This apparent dissociation is mechanistically plausible because somatic voltage-clamp measurements primarily reflect somatic and proximal conductance and are limited in their ability to fully control the AIS, where AP initiates and maximal dV/dt occurs (Kole et al., 2008; Hu et al., 2009; Jenkins and Bender, 2025). Thus, modest or spatially restricted changes in Na^+^ channel availability at the AIS can measurably alter AP threshold, max dV/dt, and width even when somatic peak step-evoked current changes are smaller, particularly in medial neurons (Bean, 2007; Leão et al., 2008; Kim et al., 2019). In addition, AP waveform is a nonlinear readout shaped by the timing of Na^+^ recruitment and its interplay with repolarizing K^+^ conductance. In fast-spiking auditory brainstem neurons, K_*V*_ currents including K_*V*_3 channels strongly constrain spike width, so small Na^+^ perturbations can be amplified into detectable waveform changes through altered Na–K interplay (Wang et al., 1998; Rudy and McBain, 2001). Finally, the pronounced I_*NaP*_ reduction in lateral neurons provides another clue to altered near-threshold dynamics, precisely the voltage range that most strongly influences spike initiation and early trajectory (Crill, 1996), helping reconcile why lateral neurons show the clearest current phenotype while both regions show waveform sensitivity during early development. Future studies combining spatially resolved transcriptomics (e.g., RNA-scope) with compartment-specific labeling would be well suited to test whether tonotopic differences in excitability arise from graded expression of Na_*V*_ and K_*V*_ channel subtypes within distinct subcellular domains of MNTB principal neurons, particularly the soma versus the AIS, and to determine whether such molecular gradients preferentially emerge in the lateral (low frequency) MNTB during the pre-hearing window.

### 4.2 *Scn2a* haploinsufficiency is not sufficient to increase MNTB firing activity

*Scn2a* haploinsufficiency is known to play a significant role in neuronal hyperexcitability in the striatum (Zhang et al., 2021), hippocampus (Ogiwara et al., 2018), and neocortex (Spratt et al., 2021; Zhang et al., 2021). We also expected that *Scn2a* haploinsufficiency might increase intrinsic excitability and spike output in the MNTB, however, our findings show similar AP numbers in *Scn2a^+/+^* and *Scn2a^+/–^* mice (Figure 5). One possible explanation is due to the specialized operating regime of MNTB principal neurons, which are optimized for phasic, time-locked spiking and high-fidelity transmission rather than sustained tonic firing during prolonged depolarization (Forsythe, 1994; Borst and Soria van Hoeve, 2012). In this context, spike-count measurements during square current steps can have limited dynamic range and may be relatively insensitive to moderate reductions in Na^+^ conductance. Preservation of spike output is also consistent with developmental homeostatic mechanisms that stabilize excitability via compensatory adjustments in other conductance. For example, the large contribution of K_*V*_ channel, especially K_*V*_1.1 and K_*V*_1.2 conductance, in maintaining a single action potential following a depolarization pre-presynaptic spike (Dodson et al., 2002, 2003; Brew et al., 2003; Kopp-Scheinpflug et al., 2003). We attempted to reveal potential compensatory changes in intrinsic excitability by partially blocking TEA-sensitive K^+^ currents using a low concentration of TEA (1 mM). However, TEA did not increase spike output in MNTB principal neurons, and AP waveform and other intrinsic membrane properties were not substantially altered under these conditions (Supplementary Figure 6). Future studies could more selectively target specific K^+^ channel families implicated in MNTB firing (e.g., K_*V*_1- or K_*V*_3-mediated currents) using subtype-specific pharmacology and/or genetic approaches.

### 4.3 Physiological relevance of *Scn2a* expression in MNTB neurons

Hyperexcitability has been reported in other ASD-related models within the auditory brainstem. For example, in *Fmr1* mutant mice, MNTB principal neurons exhibit increased firing during sustained depolarization, which has been attributed to altered high- and low-threshold K^+^ conductance, including K_*V*_3 and K_*V*_1 mediated currents (El-Hassar et al., 2019). Importantly, pharmacological modulation of K_*V*_ channels can rescue this enhanced firing phenotype (El-Hassar et al., 2019). In contrast, we did not observe increased spike output in MNTB neurons from *Scn2a*^+/–^ mice during prolonged current injections. Although our study did not directly interrogate K_*V*_ channel kinetics or expression, one plausible interpretation is that *Scn2a* haploinsufficiency alone is insufficient to drive tonic hyperexcitability in this circuit. A different interpretation might be that compensatory adjustments in other conductance including K_*V*_ currents stabilize firing output and mask genotype-dependent effects on spike number. Future work that directly quantifies K_*V*_ channel expression and their developmental regulation in MNTB neurons from *Scn2a*^+/–^ mice will be important for identifying candidate homeostatic mechanisms.

Importantly, the physiological relevance of our findings may therefore lie less in tonic firing output and more in temporal fidelity, which includes spike latency, jitter, recovery from inactivation, and reliability during high-frequency synaptic drive from the calyx of Held, features that are exquisitely sensitive to Na^+^ channel composition and availability at the AIS. Specifically, our tonotopic results raise the possibility that Na_*V*_1.2 plays a disproportionately important role at the AIS during early postnatal development in lateral MNTB neurons. These results occur prior to maturation and/or isoform redistribution [e.g., increased Na_*V*_1.6 contribution (Boiko et al., 2001, 2003; Hu et al., 2009), Supplementary Figure 2] stabilizes peak currents after hearing onset. The current model predicts that *Scn2a* reduction would preferentially degrade spike timing precision in low-frequency circuits during a defined developmental window.

## Data Availability

The original contributions presented in this study are included in this article/Supplementary material, further inquiries can be directed to the corresponding author.
