# Supplementary material for: Scn2a, encoding NaV1.2 channel, contributes to tonotopic maturation of spike kinetics in developing mouse MNTB
Source: Front Cell Neurosci. 2026 Jun 16;20:1819425. doi: 10.3389/fncel.2026.1819425 (PMC13314488; doi:10.3389/fncel.2026.1819425)
Supplement: Supplementary file 1 [file Supplementary_file_1.docx]

Supplementary Material

# Supplementary Figures

**
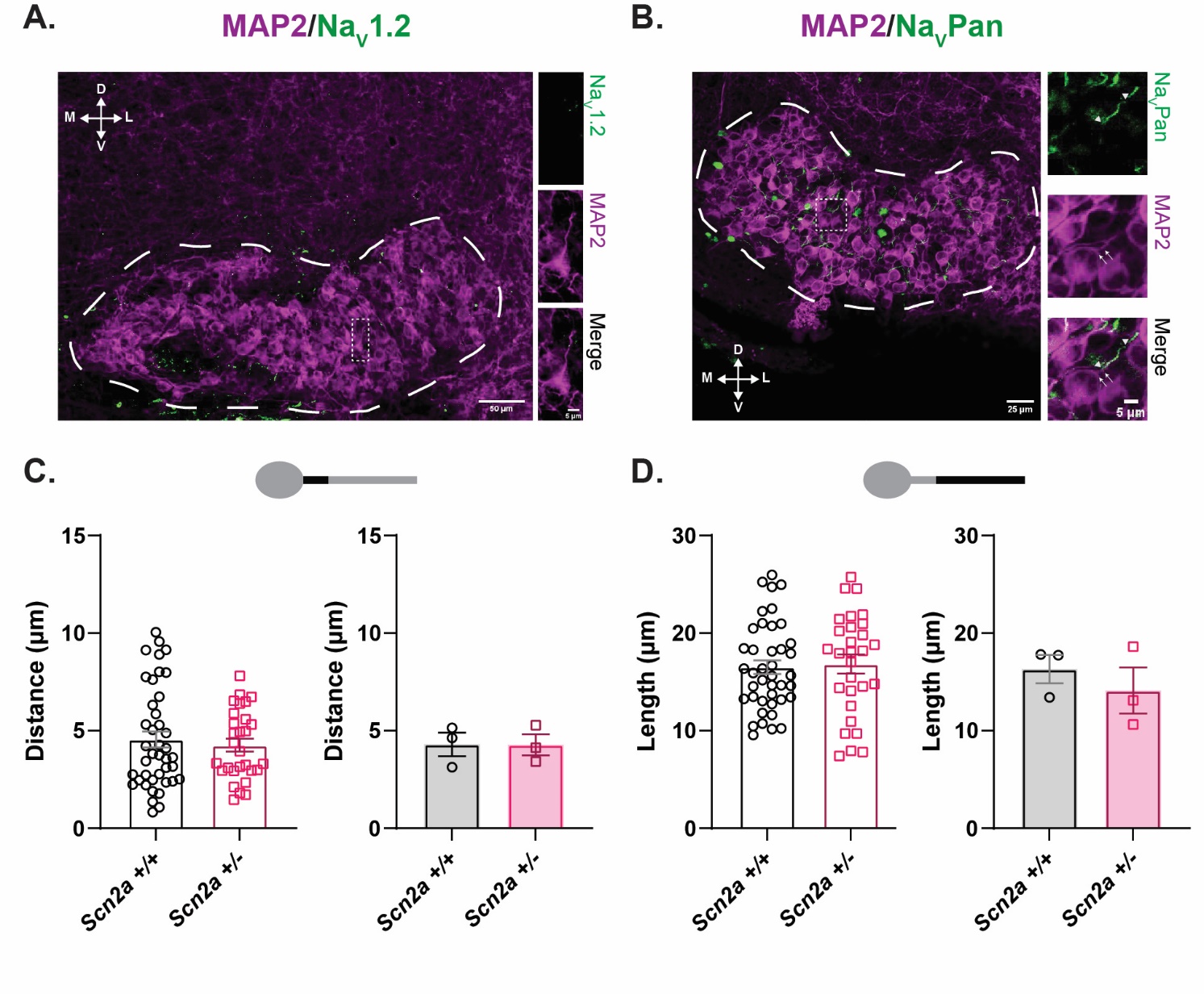
Supplementary Figure 1.** **Na*_V_*1.2 and Na*_V_*Pan immunolabeling in the MNTB and quantification of AIS geometry during the pre-hearing stage.** **(A)** Representative confocal image of the MNTB immunostained for MAP2 (magenta) and Na*_V_*1.2 (green). The MNTB is outlined with a dashed line. Higher-magnification views (right) show the Na*_V_*1.2 channel, MAP2, and merged channels in the boxed region. Under these staining conditions, Na*_V_*1.2 signal in MNTB neurons/AIS domains was not reliably detected despite clear neuronal labeling by MAP2. **(B)** Immunostaining using a pan–Na*_V_* antibody (Na*_V_*Pan; green) and MAP2 (magenta) robustly labels axon initial segments (AIS) in MNTB neurons (example AIS indicated in the boxed region; higher magnification at right shows Na*_V_*Pan, MAP2, and merged channels). Inset, a single MNTB neuron with arrows locating the distance from the soma to the start of the Na^+^ channels. Arrowheads depict the length of Na*_V_*Pan aggregation. **(C)** Quantification of axonal distance (µm) measured from MAP2 labeling of the soma to the start of Na^+^ channels in *Scn2a^+/+^* and *Scn2a^+/–^* MNTB principal neurons. Left, individual neurons; right, biological replicates, means summarized per animal. **(D)** Quantification of AIS position (length; µm) from Na*_V_*Pan labeling in *Scn2a^+/+^* and *Scn2a^+/–^* MNTB principal neurons. Data are presented as mean ± SEM. There was no difference in distance or length.

**
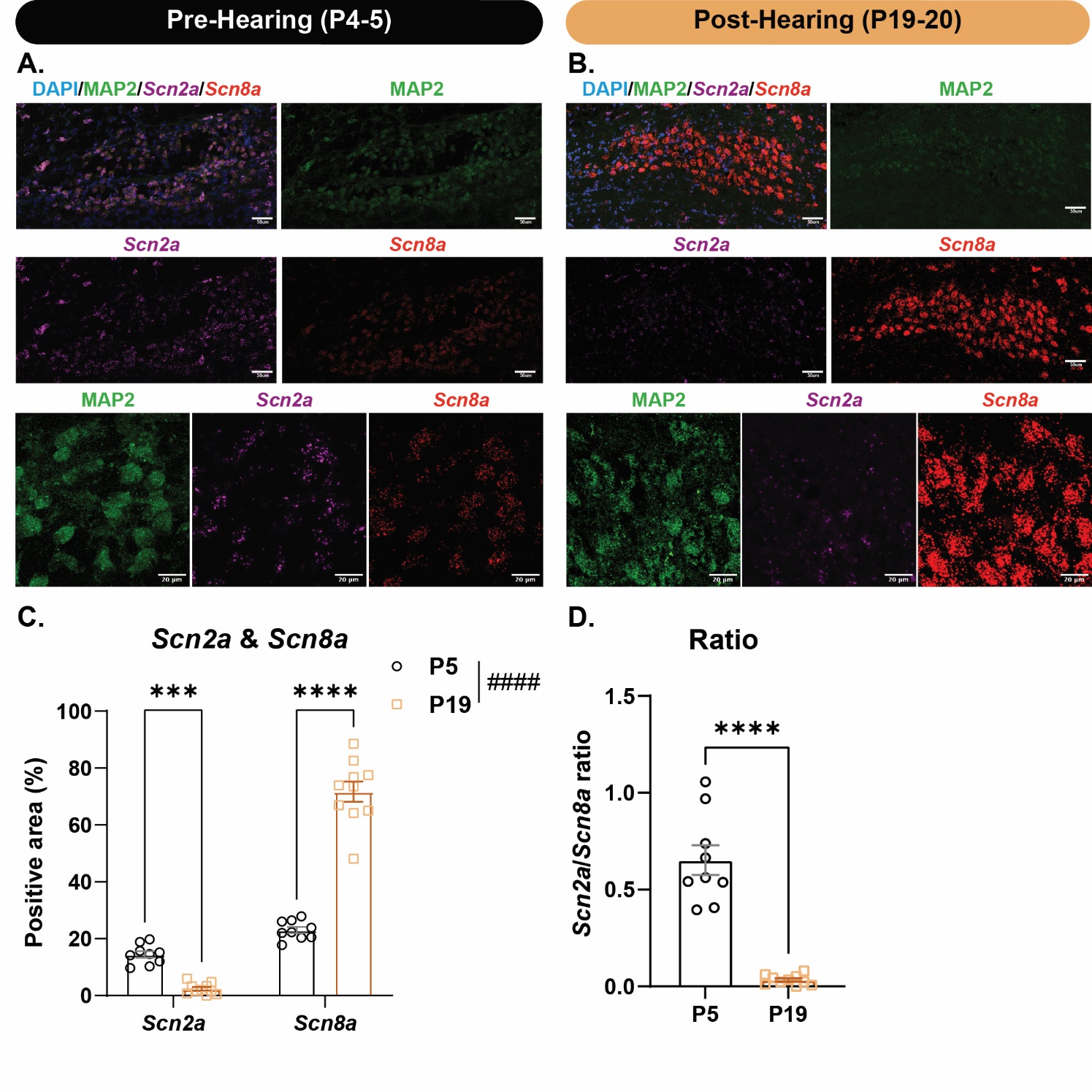
**

**Supplementary Figure 2.** **Fluorescent in situ hybridization (FISH) shows high expression of *Scn2a* at the pre-hearing stage but *Scn2a* expression is significantly reduced at the post-hearing stage.** **(A)** Images of MNTB were taken with low magnitude objective lens (20x, above 4 images) and high magnitude objective lens (40x, below 3 images) from P4-5 **(A)** and P19-20 **(B)**. Transcripts of *Snc2a* and *Scn8a* were labeled as magenta and red respectively, and protein MAP2 was labeled as green. **(C)** To quantify the expression of each gene, area with positive signal of each channel was measured within each cell (MAP2 positive area, *n* = 9 cells for P4-5 group, *n* = 10 cells for P19-20 group). Two-way ANOVA test suggests significant difference between age (*p* < 0.0001). Higher expression of *Scn2a* in P4-5 (*p* = 0.0004) than P19-20 (*p* < 0.0001) and lower expression of *Scn8a* in P4-5 than P19-20. **(D)** To evaluate relative expression *Scn2a* along with age, ratio of *Scn2a*/*Scn8a* was calculated and compared between two age groups. Consistently, the relative expression of *Scn2a* was significantly higher at P4-5 than P19-20. Data are presented as mean ± SEM. ^####^*p* < 0.0001 based on two-way ANOVA. ****p* < 0.001, *****p* < 0.0001 based on multiple comparison test with Bonferroni’s correction.


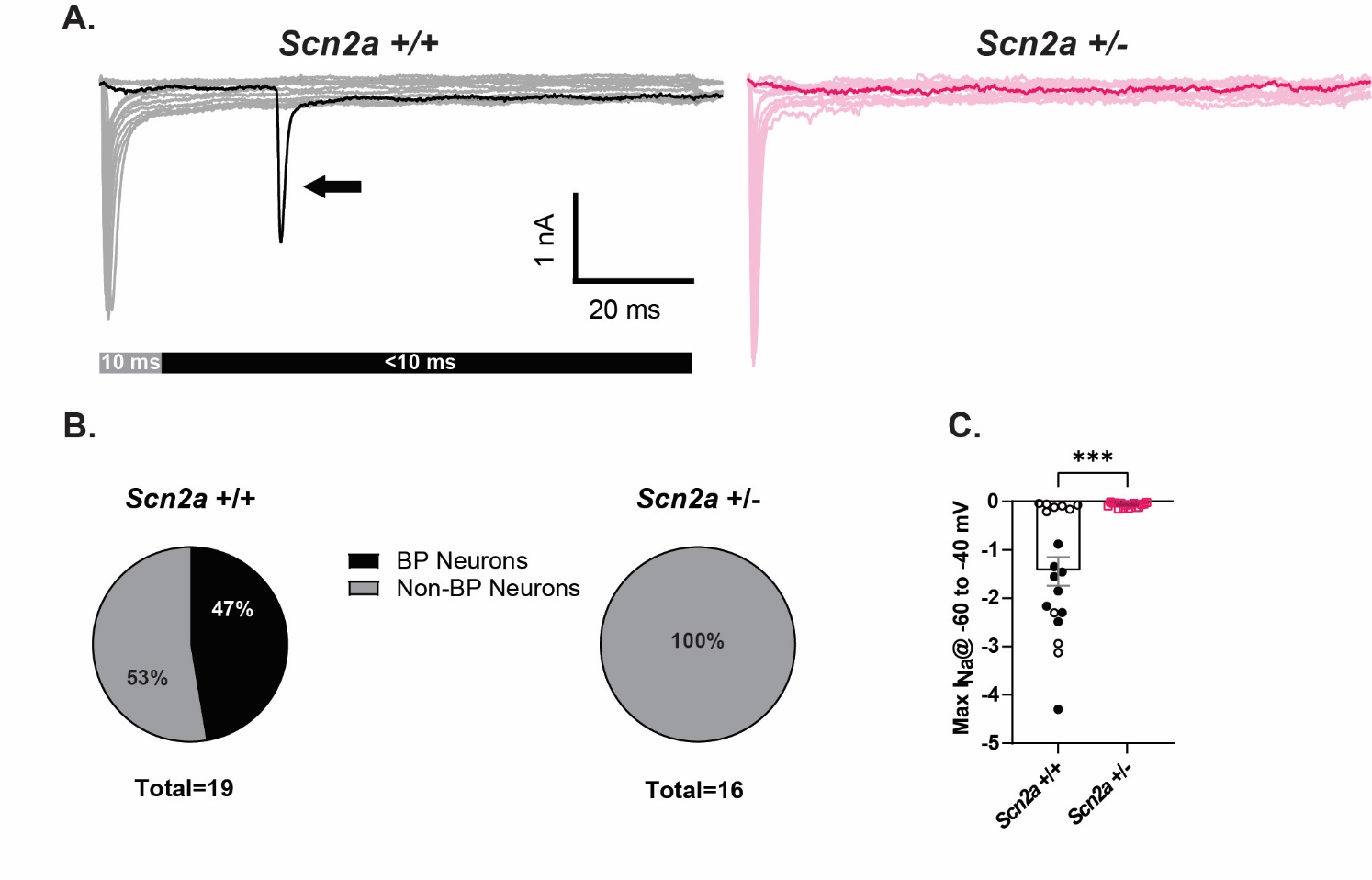


**Supplementary Figure 3.** **Loss of a time-delayed (“backpropagating”) Na^+^ current component in pre-hearing *Scn2a^+/–^* MNTB principal neurons.** **(A)** Representative I*_Na_* evoked by a depolarizing voltage-step protocol in pre-hearing MNTB principal neurons from *Scn2a^+/+^* (black) and *Scn2a^+/–^* (pink) mice. In a sub-population of *Scn2a^+/+^* neurons, the current waveform includes a delayed-onset inward component (arrow/marked trace), consistent with recruitment of electrotonically remote axonal Na*_V_* conductance that are imperfectly controlled under somatic voltage clamp (i.e., a “backpropagating” component). First 10 ms marks the timepoint used for transient I*_Na_* measurements. Scale bars: 1 nA, 20 ms. **(B)** Proportion of neurons exhibiting the delayed/backpropagating Na^+^ current component (“BP neurons,” black) versus neurons lacking this component (“Non-BP neurons,” gray). In *Scn2a^+/+^* mice, 47% of neurons were classified as BP (9/19 cells) and 53% as non-BP (10/19 cells). In *Scn2a^+/–^* mice, no neurons exhibited the BP component (0/16 cells; 100% non-BP). **(C)** Summary of the maximal amplitude of the delayed/backpropagating Na^+^ current component in *Scn2a^+/+^* versus *Scn2a^+/–^* neurons, demonstrating a marked reduction/absence in *Scn2a^+/–^* cells (*p* = 0.0002). Individual neurons are plotted with group summary overlaid. Filled circles mark the MNTB principal neurons exhibiting the time-delayed inward Na^+^ current component. Data are presented as mean ± SEM. ****p* < 0.001 based on Unpaired *t*-test.


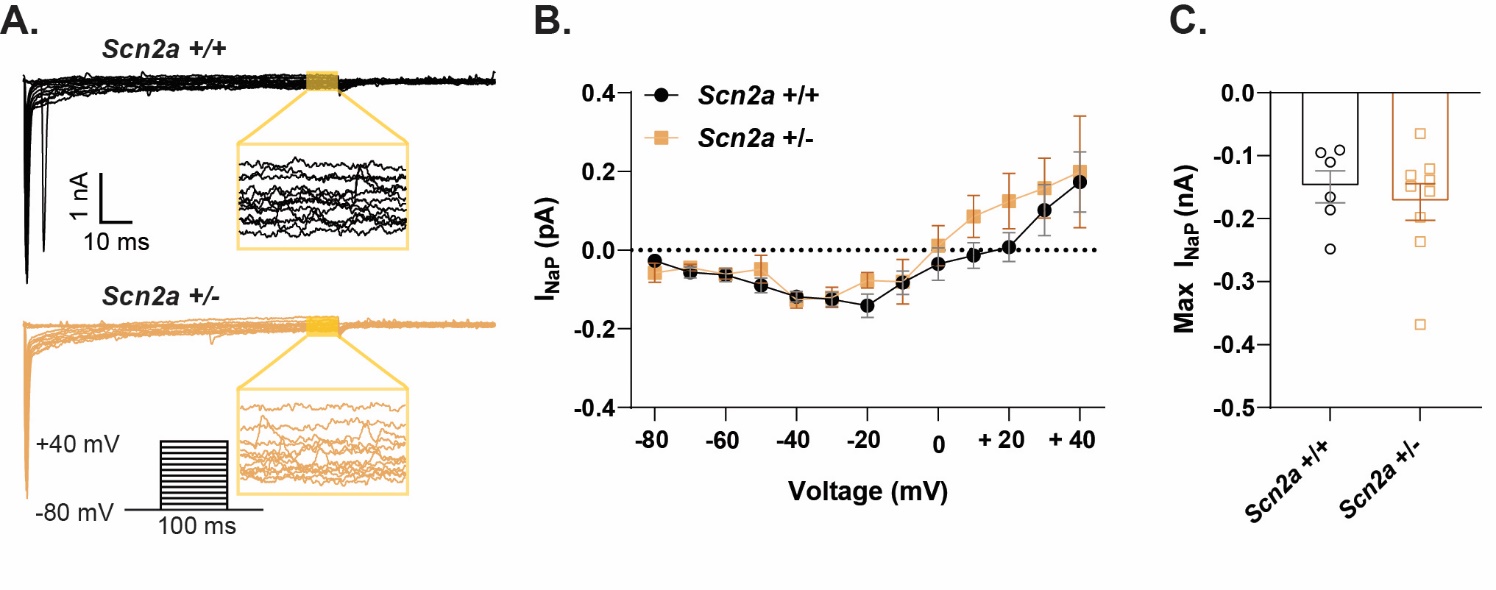
**Supplementary Figure 4.** **I*_NaP_* was not changed in MNTB neurons from *Scn2a^+/–^* mice in the post-hearing period.** **(A)** Representative traces for I*_NaP_* in *Scn2a^+/+^* (top, black) and *Scn2a^+/–^* (bottom, orange) MNTB principal neurons from P14-19. **(B)** I-V relationship for I*_NaP_* comparing genotypes across command potentials. **(C)** Summary of max I*_NaP_* amplitude in *Scn2a^+/+^* and *Scn2a^+/–^* MNTB principal neurons. Individual neurons are plotted with group summary overlaid (mean ± SEM). There was no difference.


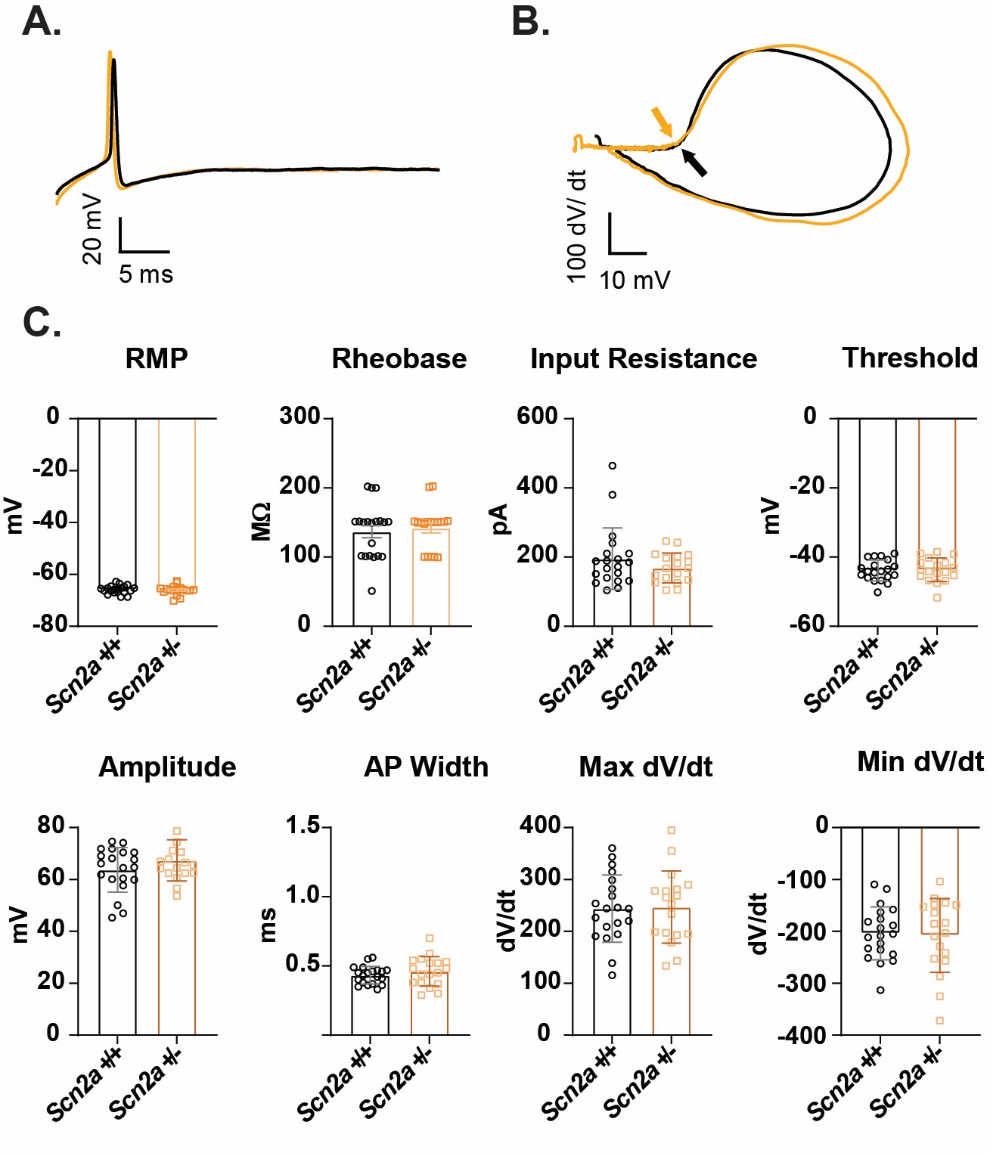


**Supplementary Figure 5.** **Action potential waveform and intrinsic membrane properties are largely preserved in *Scn2a^+/–^* MNTB principal neurons in the post-hearing stage.** **(A)** Representative single action potentials (APs) recorded in whole-cell current clamp from MNTB neurons of *Scn2a^+/+^* (black) and *Scn2a^+/–^* (orange) mice. **(B)** Representative phase plots (dV/dt vs membrane potential) from MNTB neurons have similar AP initiation and waveform kinetics in *Scn2a^+/–^* compared to *Scn2a^+/+^* neurons. Arrows indicate differences in AP initiation/trajectory near threshold and during the upstroke/repolarization. **(C)** Summary of intrinsic and AP waveform properties measured from MNTB neurons, including RMP, rheobase, input resistance, AP threshold, AP amplitude, AP width, maximal upstroke slope (max dV/dt), and maximal repolarization slope (min dV/dt). Data are presented as mean ± SEM.


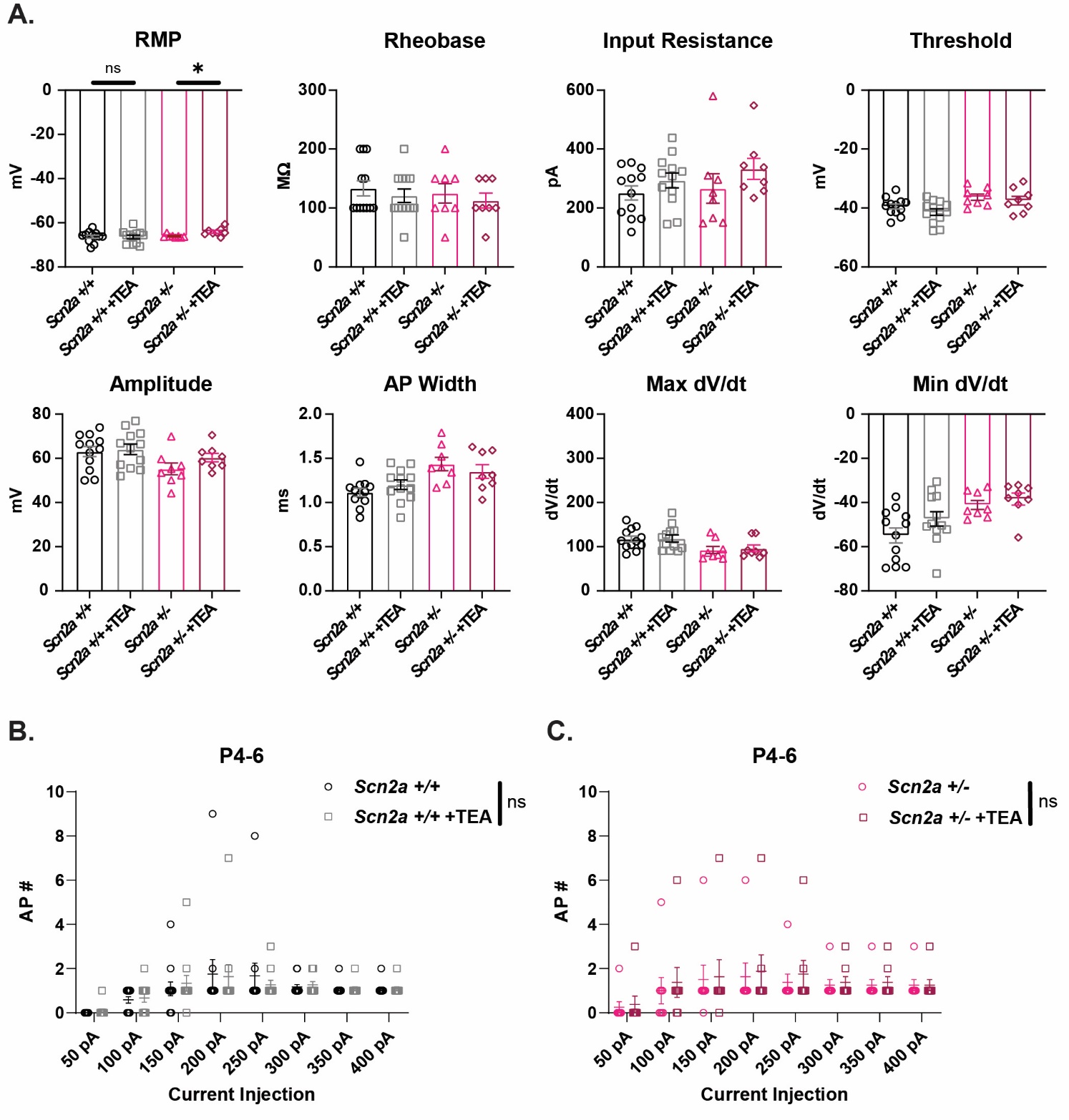


**Supplementary Figure 6.** **Low voltage potassium channel blockage preserves action potential intrinsic membrane properties and firing in *Scn2a^+/–^* MNTB principal neurons. (A)** Summary of intrinsic and AP waveform properties in MNTB principal neurons treated with low-voltage potassium blocker, TEA (1mM). Only RMP was reduced in presence of TEA (1mM) in *Scn2a^+/-^* MNTB neurons (*p* = 0.038). Rheobase, input resistance, AP threshold, AP amplitude, AP width, maximal upstroke slope (max dV/dt) and maximal repolarization slope (min dV/dt) had no effect in the presence of TEA (1mM). **(B)** Action potential number comparison in *Scn2a^+/+^* or **(C)** *Scn2a^+/-^* without or in presence of TEA (1mM) in MNTB neurons. Data are presented as mean ± SEM. **p* < 0.05 based on Mann-Whitney *U* test.
